# Development and Preclinical Application of an Immunocompetent Transplant Model of Basal Breast Cancer with Lung, Liver and Brain Metastases

**DOI:** 10.1371/journal.pone.0155262

**Published:** 2016-05-12

**Authors:** Olga Aprelikova, Christine C. Tomlinson, Mark Hoenerhoff, Julie A. Hixon, Scott K. Durum, Ting-hu Qiu, Siping He, Sandra Burkett, Zi-Yao Liu, Steven M. Swanson, Jeffrey E. Green

**Affiliations:** 1 Laboratory of Cancer Biology and Genetics, National Cancer Institute, NIH, Bethesda, Maryland, United States of America; 2 Laboratory of Molecular Immunoregulation, NCI, NIH, Frederick, Maryland, United States of America; 3 Comparative Molecular Cytogenetics Core, Frederick National Laboratory for Cancer Research, NCI, Frederick, Maryland, United States of America; 4 School of Pharmacy, University of Wisconsin-Madison, Madison, Wisconsin, United States of America; University of Tennessee Health Science Center, UNITED STATES

## Abstract

Triple negative breast cancer (TNBC) is an aggressive subtype of breast cancer that is associated with a poor prognosis and for which no targeted therapies currently exist. In order to improve preclinical testing for TNBC that relies primarily on using human xenografts in immunodeficient mice, we have developed a novel immunocompetent syngeneic murine tumor transplant model for basal-like triple-negative breast cancer. The C3(1)/SV40-T/t-antigen (C3(1)/Tag) mouse mammary tumor model in the FVB/N background shares important similarities with human basal-like TNBC. However, these tumors or derived cell lines are rejected when transplanted into wt FVB/N mice, likely due to the expression of SV40 T-antigen. We have developed a sub-line of mice (designated REAR mice) that carry only one copy of the C3(1)/Tag-antigen transgene resulting from a spontaneous transgene rearrangement in the original founder line. Unlike the original C3(1)/Tag mice, REAR mice do not develop mammary tumors or other phenotypes observed in the original C3(1)/Tag transgenic mice. REAR mice are more immunologically tolerant to SV40 T-antigen driven tumors and cell lines in an FVB/N background (including prostate tumors from TRAMP mice), but are otherwise immunologically intact. This transplant model system offers the ability to synchronously implant the C3(1)/Tag tumor-derived M6 cell line or individual C3(1)/Tag tumors from various stages of tumor development into the mammary fat pads or tail veins of REAR mice. C3(1)/Tag tumors or M6 cells implanted into the mammary fat pads spontaneously metastasize at a high frequency to the lung and liver. M6 cells injected by tail vein can form brain metastases. We demonstrate that irradiated M6 tumor cells or the same cells expressing GM-CSF can act as a vaccine to retard tumor growth of implanted tumor cells in the REAR model. Preclinical studies performed in animals with an intact immune system should more authentically replicate treatment responses in human patients.

## Introduction

The immune system plays a critical role in cancer biology and response to therapy, yet the most commonly used preclinical models of mammary cancer utilize immunodeficient hosts into which human xenografts are transplanted [1, 2]. The lack of an intact immune system alters the tumor microenvironment such that these models less accurately recapitulate biologic processes that are critically important in human breast cancer progression and therapeutic responses. Athymic nude mice lack a normal thymus and functionally mature T cells, severe combined immunodeficient (SCID) mice have abnormal B and T cells, non-obese diabetic (NOD)-SCID mice have impaired B and T cells and deficient NK cells, and NSG mice are very immune deficient lacking B, T and NK cells as well as having defective innate immunity and abnormal cytokine signaling, which allows for functional engraftment of a variety of tumor cells [3]. Immune responses may influence signaling in the microenvironment that promote or inhibit tumor cell growth [4]. While the immune system serves to recognize and eliminate transformed cells, tumor variants with decreased immunogenicity or that activate immune checkpoint systems may evade the immune response, promoting the outgrowth of selected subsets of transformed cells [5]. Therefore, interpretation of tumor endpoints in immunocompromised mouse models may be significantly confounded by abnormalities in their immune function.

Another limitation of xenograft models is related to the stromal components derived from the host mouse that contribute to tumor growth in human xenograft models [6]. Since tumor cell-stromal cell interactions are of critical importance in determining tumor biology [7], human xenograft interactions with the mouse stroma may not replicate the same stromal-epithelial cross-talk that occurs in tumors of patients. Although patient-derived xenograft (PDX) models initially contain both human tumor and stromal cells, the human stromal component is replaced by murine stromal cells after several passages in mice [8]. In addition to the lack of an intact functional immune system, PDX models are limited by high cost, intensity of labor, and technical challenges.

Immunocompetent syngeneic mouse models have been utilized to establish organ-specific metastasis models by several rounds of transplantation, metastasis formation and re-implantation leading to the selection of cell lines that are highly metastatic [7, 9]. For example, 4T1 cells, which were originally derived from a spontaneous mouse mammary tumor arising in a BALB/C mouse, grow rapidly when injected into the fat pad of a syngeneic animal and metastasize to lungs, liver, bone, and brain [9, 10]. Although this model is highly malignant, the tumors themselves are poorly immunogenic. Due to the aggressiveness of the tumors in this model, study periods are fairly short, limiting the application of extended treatment strategies, especially related to immunotherapies [11, 12].

The C3(1)-SV40 T/t-antigen (C3(1)/Tag) transgenic mouse model of mammary carcinoma develops invasive tumors that share important molecular and biologic features with human basal-like triple-negative breast cancer (TNBC) [13–15]. SV40 Tag functionally inactivates both p53 and pRb whose functions are compromised in most human TNBCs. Genomic characterization of the tumors has revealed that they cluster closely with human basal-like breast cancers [13]. C3(1)/Tag tumors develop independently of hormone supplementation or pregnancy [16]. Female mice develop multifocal mammary hyperplasias by 3 months of age, progressing to mammary adenocarcinomas by 6 months of age in 100% of the animals. These tumors exhibit histologic characteristics of aggressive human invasive ductal carcinomas [15]. This model has been widely used for pre-clinical testing of various preventive and therapeutic compounds [17, 18].

Although the C3(1)/Tag model has proven highly useful for understanding breast cancer biology and for pre-clinical testing, it is limited by the fact that multiple tumors generally develop in the mice over a 3- to 6-month time-period requiring euthanasia prior to the development of robust lung metastatic lesions. In our experience, C3(1)/Tag tumors and our mammary tumor-derived cell lines can not be transplanted into wild type FVB/N mice since they are rejected likely due to the expression of the SV40 viral oncoprotein Tag. However, a previous study reported the development of cell lines developed from C3(1)/Tag mammary tumors that could be grown in wild-type FVB/N mice [19]. In order to overcome these potential immunologic limitations of transplanting Tag-derived tumors into FVB/N mice based upon our studies, we have developed a line of mice (C3(1)/Tag-REAR) that appears tolerant to Tag allowing for the transplant of Tag-expressing tumors and/or cell lines in the FVB/N background.

The C3(1)/Tag-REAR sub-line spontaneously arose from the original C3(1)/Tag founder line through the loss of the original multiple copies of the C3(1)/Tag transgene in the founder line. REAR mice do not spontaneously develop a cancer phenotype, are immunologically tolerant to cells expressing the SV40 T-antigen including tumors from the C3(1)/Tag and TRAMP models, but have a normally functioning immune system as evidenced by a normal complement of sub-classes of immune cells and the rejection of human tumor cells and murine tumors derived from a non-FVB/N background. We further demonstrate that vaccination of REAR mice with irradiated C3(1)/Tag tumor cells with or without the expression of granulocyte-macrophage colony-stimulating factor (GM-CSF) significantly inhibits growth of C3(1)/Tag-derived mammary fat pad tumors, demonstrating that induction of an anti-tumor immune response can be elicited in this model. Therefore, this model system should be useful for immunotherapy studies directed against basal-like TNBC.

C3(1)/Tag tumors or cell lines can be implanted synchronously into the mammary fat pads of large cohorts of immunocompetent REAR mice. This bypasses the need to wait for tumors to develop spontaneously in multiple transgenic mice over a variable time course, allowing for therapeutic interventions to be performed in a relatively short time-frame. Importantly, the transplanted tumors or tail vein injected M6 cells derived from a C3(1)/Tag tumor exhibit a high rate of metastasis to multiple organs, including the lung, liver and brain.

The C3(1)-Tag-REAR model system provides a novel immunocompetent transplant model for analyzing preclinical therapies for basal-like triple negative breast cancer.

## Materials and Methods

### Mice

Animal studies were carried out in strict accordance with the recommendations in the Guide for the Care and Use of Laboratory Animals of the National Institutes of Health in an Association for Assessment and Accredication of Laboratory Animal Care (AAALAC) approved animal facility at NCI, Bethesda. The protocol was approved by the National Cancer Institute Animal Care and Use Committee (ACUC, Protocol # LC-063). Mice were fed a standard NIH-31 rodent diet, provided with nestlets as enrichment, and monitored three times a week until tumors developed after which they were monitored daily. All mice survived until the experimental enpoint was reached when tumors approached 2 cm in diameter. Humane endpoints for euthanasia included any signs of lethargy, weakness, hunched posture, rough hair coat, pain, decreased ability to obtain food & water, rapid or labored breathing, neurological symptoms (tilted head, uneven gait, paralysis) or more than 15% decrease in body weight. Mice were euthanized if tumors approach 2 cm in diameter. All surgeries were performed under sterile conditions using isoflurane anesthesia, and all efforts were made to minimize suffering. Mice were euthanized using CO2 narcosis.

The development and screening of the C3(1)/Tag transgenic mouse model in the FVB/N background generated by our lab has been previously described [16]. A line of mice was identified that was positive for the transgene by genotyping but the animals did not develop the expected mammary cancer phenotype. This line was bred to wt FVB/N mice (Charles River, Frederick, MD) and further characterized. This subline of mice is designated the C3(1)/Tag-REAR (abbreviation for rearrangement) line.

### Transgene copy number

#### Southern Blot analysis

Genomic DNA was extracted from C3(1)/Tag and REAR mouse tail biopsies by overnight digestion in GNT-K buffer at room temperature [20]. All restriction enzyme digests were done with 15–20 units enzyme/μg DNA. Genomic DNA was digested with Sac1 and BamH1 restriction enzymes. The 7.5 kb fragment of the PRP-3 plasmid, which contains the intact transgene, was extracted with the same enzymes, releasing the C3(1) promoter-T-antigen transgene and purified as template for the probe [16]. The probe was labeled with [γ32-P] dCTP Ready–To–Go DNA labeling beads (without dCTP) (GE Healthcare Life Sciences) and purified using Illustra Probe Quant G-50 Columns (GE Healthcare Life Sciences). Digested DNA was separated on a 0.8% agarose gel and alkaline transferred to a NYTRAN Membrane (0.45 γm) using the Turbo BlotTransfer System (Schleicher & Schuell Bio Science). Pre-hybridization and hybridization of the blots were performed using QuikHyb Hybridization solution (Stratagene Products) at 65^0^ C overnight. Membranes were washed and exposed to X-ray film.

#### Quantitative PCR

Genomic DNA was extracted as above. After brief centrifugation, 2 μl of the supernatant was transferred to 10 μl PCR reactions. PCR reactions were prepared using TaqMan Universal PCR Master Mix, No AmpErase UNG (Applied Biosystems). PRP-3 plasmid (containing the T-antigen sequence) [16] was serial diluted in FVB tail lysate to generate a standard curve. Primers for amplification of an 18 bp amplicon internal to the T-Antigen sequence were 5′-GCTAATGGACCTTCTAGGTCTTGAA-3′ and 5′-AAATATGCCTTTCTCATCAGAGGAATA-3′ and the probe was 5′ FAM- CCCCAGGCACTCC-TAMRA-3′. Samples were normalized to Albumin. Primers used were Alb-forward: 5’-TTCCTGCAACACAAAGATGACA-3’ and reverse: 5’-CAGCCTCTG GCCTTTCAAAT-3’ Alb-probe: probe CCCCAGCCTGCCAC. Reactions were run in 384-well format in an ABI Prism 7900HT with the following conditions: 95°C for 1 min, followed by 95°C for 15 s, 60°C for 1 min repeated for 40 cycles.

### Fluorescence *in situ* hybridization (FISH) analysis

The 7.5-kb fragment containing the C3(1)/Tag fusion was excised from the PRP-3 plasmid and purified. Linearized DNA was tagged with Digoxigenin 11 dUTP. Dig labeled C3(1)/Tag was hybridized to metaphase slides prepared from C3(1)/Tag, C3(1)/Tag-REAR, and FVB/N primary splenic preparations and detected with anti-dig Rhodamine. The integration site was confirmed using MMU WCP (Mouse whole chromosome paint) 6 labeled in Biotin 16 dUTP detected green with AV FITC.

### Immunophenotyping and FACS analysis

#### Cell surface staining

To evaluate the expression of cell surface antigens, single cell suspensions were prepared from spleen, thymus, and lymph node of REAR or FVB/N mice by mechanical disruption using frosted slides. Bone marrow was isolated from femurs by flushing with a 22G needle attached to a 3 ml syringe. Red blood cells were lysed by incubation with ACK lysing buffer (Lonza, Walkersville, MD, USA). Cells were treated with purified anti-CD16/CD32 (2.4G2) to block Fc receptors. T-memory cells were evaluated by staining with rat anti-mouse CD4-FITC, rat anti-mouse CD8-PE and, rat anti-mouse/human CD44-PerCP. To evaluate B-cells, cells were stained with rat anti-mouse CD45R/B220-PE and rat anti-mouse IgM-FITC. NK and NKT cells were evaluated by staining with CD94-PE, NK1.1-APC and CD3-PerCP-Cy5.5. To evaluate the T-cell receptor subsets, cells were stained with γδ-TCR-PE and TCR-β PE-Cy5. Cells were fixed in 1% paraformaldehyde in PBS and analyzed using a LSR I flow cytometer and CellQuest software. Post acquisition analysis was performed using FCS Express Version 3 Software (De Novo Software). Antibodies: Purified anti-CD16/CD32 (2.4G2), Phycoerythrin (PE) rat anti-mouse CD8, rat anti-mouse CD45R/B220, hamster anti-mouse -γδT-cell receptor, Fluorescein Isothiocyanate (FITC) rat anti-mouse IgM, rat anti-mouse CD4, Phycoerythrin-Cyanine 5 (PE-Cy5) hamster anti-mouse TCR-β chain were purchased from BD PharMingen (San Diego, CA, USA). Peridinin Chlorophyll Protein-Cyanine 5.5 (PerCP-Cy5.5) rat anti-mouse/human CD44 was purchased from eBiosciences (San Diego, CA, USA). PE rat anti-mouse CD94, allophycocyanin (APC) mouse anti-mouse NK1.1 (eBiosciences San Diego, CA, USA), and hamster anti-mouse CD3 PerCP-Cy5.5 (BD PharMingen San Diego, CA, USA) were a generous gift from Dr. Steve Anderson (Frederick National Lab, Frederick, MD, USA).

### Cell lines

The mammary carcinoma cell line M6 derived from a C3(1)/Tag mammary tumor [21] was maintained in DMEM medium, high glucose (Gibco), supplemented with 5% FBS, penicillin/streptomycin and sodium pyruvate (Invitrogen). For cell vaccination studies, M6 cells were transduced with pBabe-GM-CSF to generate M6 cells expressing mouse GM-CSF. Human breast adenocarcinoma MDA-MB-231 cells (ATCC) were maintained in RPMI-1640 (Gibco), supplemented with 5% FBS, L-glutamine and Pen/Strep. The mouse mammary tumor cell lines 4T1 (derived from a spontaneous mammary tumor in a Balb/c mouse) [9], transgenic mammary tumor cell lines 6DT1 [22] and Myc83 [23] (derived from MMTV-cMyc transgenic mammary tumors), and MCF10A-Ras cells [24] were cultured in DMEM supplemented with 10% FBS, sodium pyruvate and 1% Pen/Strep.

### *In vivo* tumorigenicity assay

All cells were washed and resuspended in PBS at a density of 1 x 10^6^ cells in 100 μl PBS. 1 x 10^6^ cells were injected into the #4 mammary fat pads of female C3(1)/Tag-REAR mice or FVB/N mice. For lung metastasis studies 1 x 10^6^ cells were injected into the tail vein of REAR or FVB/N female mice.

#### TRAMP tumor transplantation

Prostate tumor tissues were dissected under sterile conditions from the transgenic adenocarcinoma mouse prostate (TRAMP) mouse model in the FVB/N background (FVB.B6-Tg(TRAMP)8247Ng) [25] provided by Dr.Ronald Wood, University of Rochester). Tumor tissues were kept in PBS and cut into small pieces (1–2 mm^3^ in diameter) and implanted subcutaneously into the flank of mice C3(1)/Tag-Rear and FVB/N male mice. Following implantation, tumor diameters were measured using calipers and tumor volumes were calculated using the formula: maximum diameter×(minimum diameter)^2^×0.4 [26].

### Histological and immunohistochemical analyses

Gross lesions, major organs and tissues, and the accessory sex glands were collected at necropsy and fixed in 4% (w/v) paraformaldehyde. Tissues were embedded in paraffin and sections were routinely processed and stained with hematoxylin and eosin (H&E). For immunohistochemical labeling, selected sections were deparaffinized in xylene and rehydrated through graded alcohols. Endogenous peroxides were blocked with 3% hydrogen peroxide, and antigen retrieval was performed using microwave in distilled water [27]. Following washing, sections were labeled using the DAKO ARK Kit (#K3954) per the manufacturer’s instructions. Anti-SV40 Tag mouse monoclonal antibody (BD Pharmingen # 554149) was used at a dilution of 1:25. Antibody-antigen complexes were visualized using 3,3’-diaminobenzidine chromagen (DAB) (Dakocytomation, Carpinteria, CA), and slides were counterstained with Mayer’s hematoxylin, dehydrated through graded alcohol, soaked in xylene and coverslipped.

### IFNγ ELISPOT Assay

ELISPOT assay was performed as described previously [28]. Briefly, MultiScreen-IP plates (PVDF membranes, Millipore) were coated overnight at room temperature with 50 μg/well of anti-mouse IFNγ capture antibody (Biosource International, Camarillo, CA). Effector cells at specified concentrations were added to triplicate wells followed by 5 x 10^4^ target cells per well. After effector and target cells were incubated at 37°C, the plates were washed with PBS + 0.05% Tween20 and 50 μl/well of biotinylated anti-mouse IFNγ detecting antibody (PharMingen, San Jose, CA) diluted to 1.3 μg/ml in PBS with 1%BSA and 0.05% Tween20. Plates were incubated with detecting antibody for 2 hrs at room temperature, washed 4 times with PBS followed by 1 hour incubation with Streptavidin-Alkaline Phosphatase (Gibco BRL Life Technologies). The spots were visualized with 100 μl/well of BCIP-NBT phosphatase substrate (KPL, Gaithersburg, MD) and subjected to automated evaluation using the ImmunoSpot Imaging Analyzer system (Cellular Technology Ltd, Cleveland, OH).

### Determination of GM-CSF production

GM-CSF production by M6 cells transduced by retroviral mouse GM-CSF was performed by using Mouse GM-CSF Quantikine ELISA Kit (R&D Systems) according to the manufacturer’s instruction. The results showed that 10^6^ cells produced 47 ng/ml of GM-CSF during 24 hours.

### Statistical analyses

Statistical analysis was performed using Prism version 6.0 (GraphPad software Inc., San Diego, CA). Student’s t-test and the Mann-Whitney U-test were used to compare differences between the two groups. A p-value of less than 0.05 was considered to indicate a significant difference; p values: *<0.05, **<0.01. The Pearson correlation coefficient R^2^ between tumor volume and number of cytotoxic lymphocytes in ELISPOT assay was determined using the statistical analysis provided by Excel.

## Results

### C3(1)/Tag-REAR mice underwent a spontaneous reduction in transgene copy number

Routine screening of C3(1)/Tag mice identified female mice that carried the transgene but which did not develop the expected phenotype of mammary tumors when observed for up to one year (this sub-line of C3(1)/Tag mice is designated C3(1)/Tag-REAR). In order to determine whether this was the result of a change in the original approximately 30 copies of the transgene that had integrated into one locus [16], quantitative PCR was employed as described in the Methods section. The ratio of T-antigen to albumin in genomic DNA from mouse tails was determined (S1 Fig). There was no signal amplification of Tag when genomic DNA from a wild type FVB/N mouse was used as template, or in the no template control. For C3(1)/Tag mice, the ratio was ~11:1: for REAR mice, it was ~2:1.

**Fig 1:**
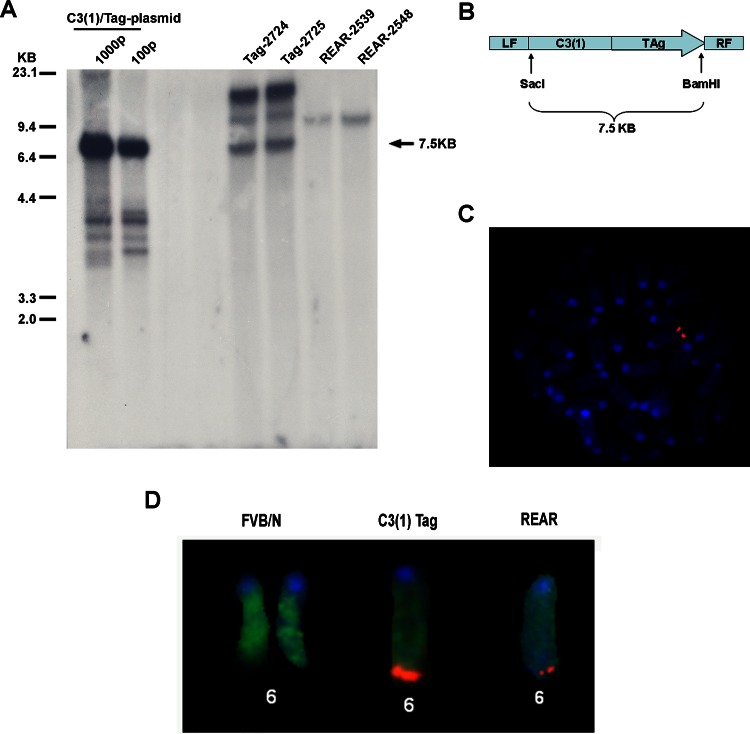
Transgene analyses. (A) Southern blot analysis of genomic DNA. SacI and BamHI digest of C3(1)Tag mouse genomic DNA (Tag-2724 and Tag 2725) and REAR mouse genomic DNA (REAR-2539 and REAR 2548) probed with the 7.5 kb C3(1)Tag transgene [16] as depicted in (B). Note that the 7.5 kb band representing the entire transgene is present in the C3(1)/Tag mice but absent in the REAR mice. However, an approximately 12 kb band representing the transgene with genomic flanking region is found in both the C3(1)/Tag and REAR mice. (C) FISH analysis of a metaphase spread of splenocytes from the original C3(1)/Tag mouse line using the C3(1)/Tag construct (B) as probe demonstrating transgene on chromosome 6 as previously reported. (D) FISH analyses of chromosome 6 from spleens of normal FVB/N mice, C3(1)/Tag mice, and REAR mice, probed with the same probe as in (C).

Additionally, Southern blot analysis revealed that a reduction in transgene copy number had taken place in the REAR mice with apparently one copy of the transgene remaining in the genome (Fig 1A). A 7.5 kb band representing the full transgene (Fig 1B) was observed only in the original C3(1)/Tag line of mice (Tag-2724 and Tag-2725) that develop the expected tumor phenotype whereas an approximately 12 kb band representing at least part of the transgene fused to the genomic flanking region was found in both the C3(1)/Tag and REAR mice (Fig 1A). The 12 kb band was the only band identified in the REAR mice suggesting that one copy of the transgene remained in the REAR mice. In order to further confirm that a significant reduction in transgene copy number occurred in the C3(1)/Tag-REAR mice, FISH analysis was performed on C3(1)/Tag mice and the C3(1)/Tag-REAR sub-line using the entire 7.5 kb transgene as probe (Fig 1C and 1D). Metaphase spreads of spleen cells demonstrated localization of the C3(1)/Tag transgene on mouse chromosome 6 (Fig 1C and 1D) as previously determined [29]. As seen in Fig 1D, a significant reduction in signal intensity was observed in the FISH analysis of C3(1)/Tag-REAR spleen cells compared to spleen cells from the C3(1)/Tag founder line of mice with no detectable signal observed in control cells from wt FVB/N mice. These results demonstrate that a rearrangement of the transgene occurred resulting in loss of most of the original transgene copies, apparently leaving only one copy or a partial copy of the transgene in the original chromosomal locus. This remaining portion of the transgene is sufficient to induce tolerance to SV40 Tag in REAR mice as demonstrated below.

### C3(1)/Tag-REAR mice do not develop tumor phenotypes

Fifty-three C3(1)/Tag-REAR mice (44 females, 9 males) were phenotyped for histopathologic abnormalities of phenotypes known to occur in the original line of C3(1)/Tag mice. The original FVB/N line of female C3(1)/Tag mice develop atypical mammary ductal hyperplasia by 2 months of age that progress to invasive adenocarcinoma usually between 16–20 weeks of age with a 15% incidence of lung metastases by 6 months of age [27]. Due to the development of mammary tumors approaching 2 cm in diameter, C3(1)/Tag mice are euthanized by 6 months of age.

**Fig 2:**
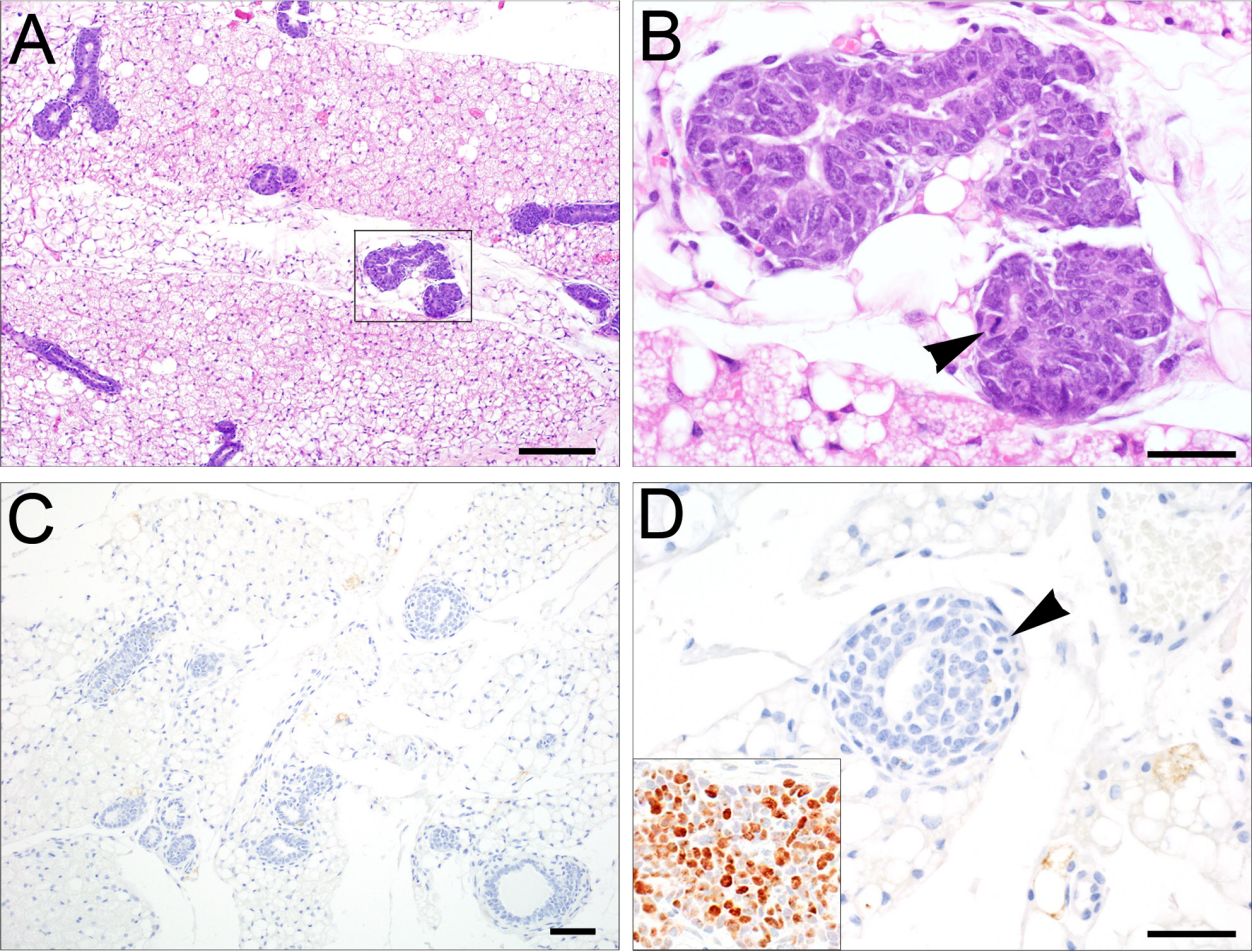
C3(1)/Tag-REAR mammary glands. (A) Minimal to mild ductal hyperplastic changes were seen (10X, Bar = 100 μm), consisting of crowding and piling of epithelial cells within glandular lumens, often with enlarged nuclei and increased mitoses. (B) higher magnification of highlighted hyperplastic lesion of A (arrowhead, 60X, Bar = 20 μm). (C) These hyperplasias were negative for the expression of SV40 T-antigen by IHC staining (20X, Bar = 100 μm). (D) higher magnification of a lesion in C (60X, Bar = 20 μm). Inset in D is a C3(1)/Tag mammary tumor as positive control for Tag staining).

Mammary tumors did not occur in C3(1)/Tag-REAR mice, except for one animal (at 19 months of age), and this tumor was negative for SV40 T-antigen. There were minimal to mild ductular hyperplasias in 14/44 females (Table 1; Fig 2) which were negative for SV40 T-antigen (Fig 2D; inset in 2D is the positive control for Tag antigen staining). Half of the mammary hyperplasias observed in the female REAR mice developed after 24 weeks of age, whereas all C3(1)/Tag female mice have already been euthanized by 24 weeks due to the development of large, invasive mammary tumors. Mammary tumors that develop in REAR mice less than 18 months of age have only been observed when M6 cells or C3(1)/Tag tumor fragments were implanted into REAR mice as described below and spontaneous tumor development in the REAR mice was not a confounding factor for the interpretation of mammary tumor analyses in REAR mice less than 18 months of age.

**Table 1:** Incidental histology findings in REAR mice.

Age	Sex/mice#	Lung	Mammay gland	Prostate
9-17wks	Females (15)	NSF	Minimal to mild hyperplasia (7)	NA
	Males (0)	NSF	NA	NSF
26-38wks	Females (16)	NSF	Minimal hyperplasia (5)	NA
	Males (2)	NSF	NA	Marked medial vascular hypertrophy (1)
1–2 years	Females (13)	Alveolar hyperplasia (1)	Minimal hyperplasia (2)	NA
		Pulmonary adenoma (3)	Mammary adenocarcinoma (1)	
		Pulmonary carcinoma (1)		
		Mammary carcinoma metastasis (1)		
	Males (7)	Pulmonary adenoma (2)	NA	Marked medial vascular hypertrophy (1)
		Pulmonary carcinoma (2)	NA	Leiomyosarcoma vascular (1)

NSF—no significant finding

NA—not applicable

Number of mice are in brackets

Male C3(1)/Tag mice develop prostatic intraepithelial neoplasia (PIN) by 5–6 months of age that may progress to adenocarcinoma by 7–8 months of age [16, 30]. In contrast, there was no evidence of prostatic epithelial hyperplasia or neoplasia in the eight male C3(1)/Tag-REAR mice examined. One male mouse at 24 months of age developed a spontaneous mesenchymal tumor arising from the tunica media of large arterioles adjacent to the urethral glands consistent with a leiomyosarcoma. This tumor was negative for SV40 T-antigen. Leiomyosarcomas have been reported at a very low incidence (~1%) in the background FVB/N strain [31].

Of the 53 animals examined, C3(1)/Tag-REAR mice developed a low incidence of spontaneous lung lesions, including alveolar hyperplasia (1 animal at 1.7yrs of age), adenoma (5 animals at 1.2-2yrs of age), and carcinoma (3 animals at 1.6–1.8yrs of age), consistent with background lesions commonly observed in aged wt FVB/N mice [31]. These lesions were negative for SV40 T-antigen by immunohistochemistry. No other significant phenotypic abnormalities were observed within the examined tissues. A summary of relevant findings is presented in Table 1.

### C3(1)/Tag-REAR mice are tolerant to SV40-Tag-induced tumors but reject human xenograft and other transgenic transplant tumors

The tumor growth potential of mammary tumors and cell lines in C3(1)/Tag-REAR mice and wt FVB/N mice were assessed through mammary fat pad implantations and tail vein injections. All mice implanted with 1 mm^3^ fragments of C3(1)/Tag mammary tumors in the mammary fat pads developed mammary tumors (5/5) whereas no tumors grew when fragments were implanted into wt FVB/N mice (Table 2).

**Table 2:** Xenograft growth of various cell lines in C3(1)/Tag-Rear Mice.

Cell line	Mouse	Mice with tumor	Cell number
Syngeneic (FVB/N)			
C3(1)Tag tumor	REAR	5/5	1mm3
6DT1	REAR	5/5	1x106
MMTV-cMyc	REAR	5/5	1x106
M6	REAR	5/5	1x106
C3(1)Tag tumor	FVB/N	0/5	1mm3
M6	FVB/N	0/5	1x106
Non-syngeneic			
4T1 (BalbC)	REAR	0/5	1x106
MCF10A-Ras (human)	REAR	0/5	3x106
MDA MB231 (human)	REAR	0/5	5x106

We also analyzed several other mammary tumor cell lines for growth in REAR mice (Table 2). Only those cell lines derived from FVB/N mice (6DT1, MMTV-cMyc, and M6) were able to grow as tumors in both the REAR and FVB/N mice. Cell lines from a non-syngeneic Balb/c mouse strain (4T1 cells) and human breast cancer cell lines (MDA-MB-231 and MCF10A-Ras cells) also did not grow in REAR mice.

**Fig 3:**
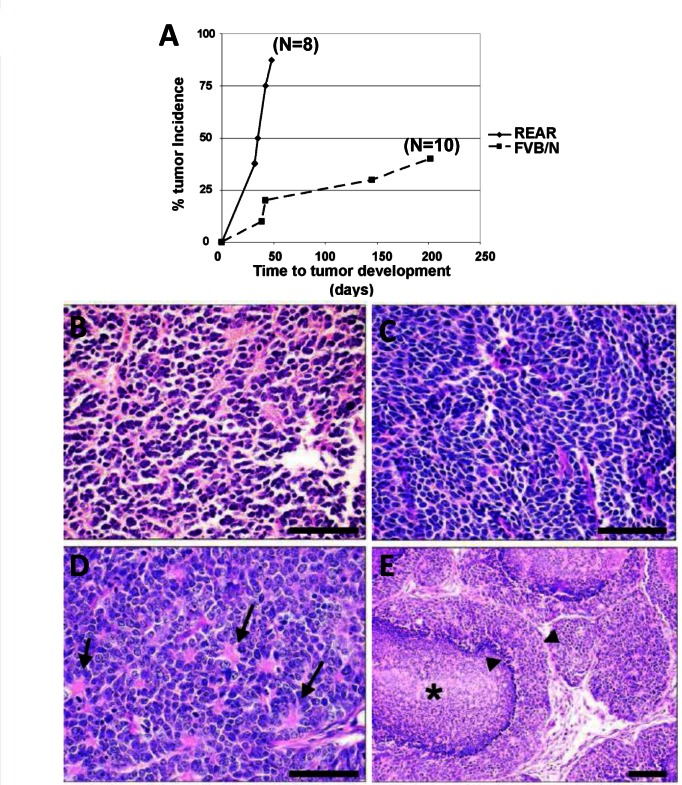
REAR mice support the growth of SV40 T-Antigen TRAMP prostate tumors. **(**A) Tumor incidence of TRAMP tumors subcutaneously transplanted into the flank region of male REAR and wt male FVB/N mice. 7/8 (87.5%) male REAR mice developed TRAMP tumors by 7 weeks following implantation. However, 4/10 FVB/N mice implanted with TRAMP tumors developed tumors at a much slower rate taking up to 7 months for tumor development. (B) Subcutaneous xenografts of TRAMP tumors were composed of solid sheets of small polygonal to ovoid neoplastic cells with scant cytoplasm and dense nuclei. (C) Tumors often exhibited neuroendocrine features including angular, carrot-shaped nuclei or (D) rosettes radiating from a centralized core of eosinophilic material (arrows). (E) Often numerous lobules of neoplastic cells exhibited central necrosis in a comedo fashion (asterisk), leaving a rim of viable tumor cells along the periphery (arrowheads, 20X, Bar = 50 μm).

In order to determine whether REAR mice demonstrated tolerance to another SV40 T-antigen driven tumor, we utilized prostate tumors arising in an FVB background from the aggressive TRAMP model of prostate cancer [25]. TRAMP tumors, which were originally developed in a different mouse background [32], arise in the mouse prostate through the expression of SV40 Tag under the probasin promoter. Freshly dissected TRAMP prostate tumor tissue pieces (1 mm^3^ FVB/N background) were implanted into the posterior flank of male REAR FVB/N mice. As shown in Fig 3A, 7/8 REAR mice (N = 8) developed tumors within 7 weeks. Subcutaneous TRAMP xenografts were characterized by a solid phenotype composed of sheets of small polygonal to ovoid basophilic cells with scant cytoplasm (Fig 3B and 3D), with approximately 25–30 mitoses/40X high power field, indicative of a highly malignant and aggressive neoplasm. Nuclei were round and dense to angular or elongate, often exhibiting a neuroendocrine-like phenotype characterized by carrot-shaped nuclei (Fig 3C) or rosettes (Fig 3D). Other tumors produced a comedocarcinoma-like phenotype, composed of multiple lobules with central necrosis and a peripheral rim of viable tumor cells (Fig 3E). Distant metastases were not detected histologically. Interestingly, 40% of FVB/N mice implanted with TRAMP tumors developed tumors, however, at a much slower rate (between about 50 to 200 days; Fig 3A).

**Fig 4:**
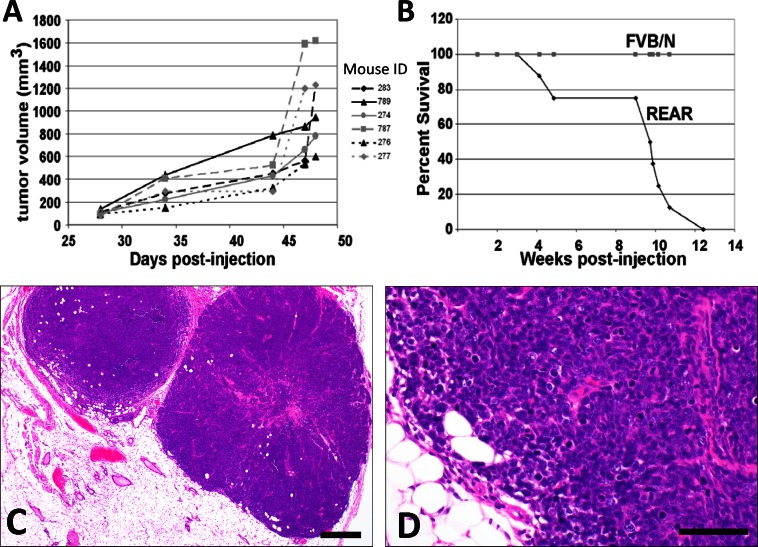
REAR mice support the growth of M6 cell line mammary tumors. (A) Growth curves of tumors arising from 1 X 10^6^ M6 cells implanted into the #2 mammary fat pad of C3(1)-REAR mice (N = 6). (B) Survival curves of REAR (N = 9) and FVB/N (N = 10) mice tail vein injected with 1 X 10^6^ M6 cells. All mice died by 12 weeks post-injection. Note that no tumors grew when M6 cells were implanted into wild-type FVB/N mice (N = 10). (C) Representative primary mammary tumor arising following the implantation of 1 X 10^6^ M6 cells into the #2 mammary fat pad (4X, Bar = 200 μm) were characterized as expansile and locally infiltrative proliferations of solid lobules and clusters of poorly differentiated epithelial cells exhibiting marked nuclear pleomorphism and atypia, and a high mitotic rate. (D) Higher magnification of C (40X, Bar = 50 μm).

1 x 10^6^ M6 cells (derived from a C3(1)/Tag transgenic tumor) implanted into the #2 mammary fat pads of REAR mice developed palpable tumors within 28 days (Fig 4A). M6 derived mammary fat pad xenograft tumors were large, solid, infiltrative mammary tumors typical of C3(1)/Tag carcinomas (Fig 4C and 4D) with variable necrosis centrally within tumors, illustrating their high growth rate. The maximum allowable tumor burden for humane considerations (approaching 2 cm in diameter) was reached by 48 days post-injection of M6 cells into the mammary fat pads at which point mice were euthanized and examined for metastases.

**Fig 5:**
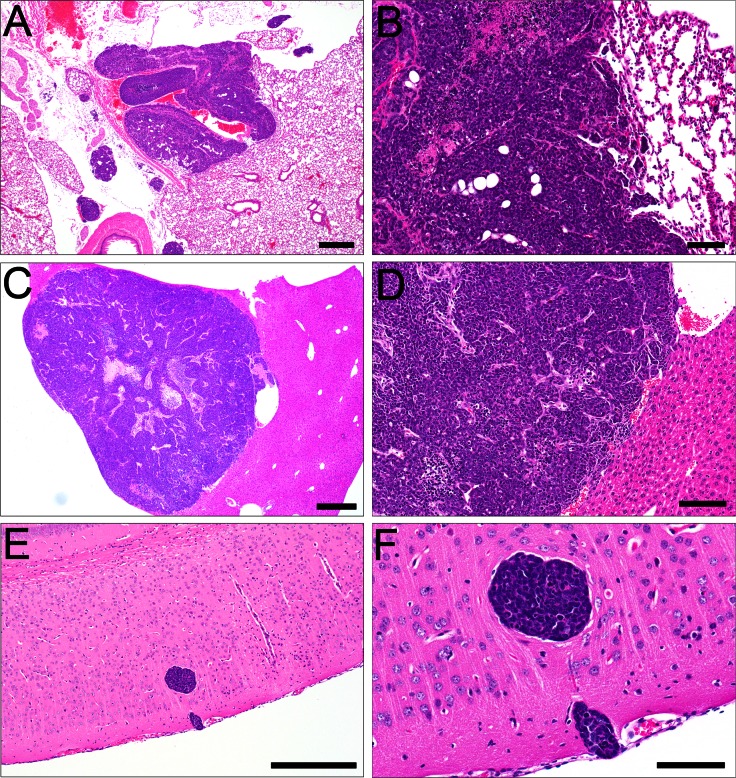
Metastatic tumors arising in REAR mice from M6 cells. (A) M6-derived mammary fat pad xenografts metastasized to the lung (4X). (B) Higher magnification of A (20X) and (C) liver (4X). (D) Higher magnification of C (10X). Tail vein M6-derived xenografts were associated predominantly with embolic growth in the lung, liver, and E) brain (10X). F) Higher magnification of brain metastasis (40X). A, C, E, Bar = 50 μm; B, D, F, Bar = 20 μm.

Metastases from M6 mammary fat pad xenografts were identified in the lung in 88% of animals (14/16) (Fig 5A and 5B) and in the liver in 27% of the animals (4/15) (Fig 5C and 5D, S1 Table). Tail vein injection of 1 x 10^6^ M6 cells into REAR mice resulted in 100% mortality by 12 weeks post-injection (Fig 4B). Growth and invasion of embolized tumor cells within the lung was observed in 100% of the mice (12/12 animals), in the liver in 67% of mice (8/12 animals), and in the brain of 45% of mice (5/11) (Fig 5E and 5F, S2 Table). The same cells implanted into the fat pad or tail-vein injected into FVB/N mice failed to develop tumors.

### C3(1)/Tag-REAR mice have normal complements of lymphoid cells

Lymphoid subsets were analyzed by flow cytometry in REAR mice and compared to FVBN mice. No significant differences in major lymphocyte subsets were detected in thymus, spleen, lymph nodes or bone marrow (Table 3). These subsets included CD4+, CD8+, CD4+CD8+, αβ+ and γδ+ T cells, NKT cells, NK cells and B cells. Thus, the failure of REAR mice to reject M6 cells was not attributable to a general lymphoid deficiency as one would observe in particular immune deficient mice, but is consistent with tolerance to the SV40T antigen that is endogenously expressed in these mice.

**Table 3:** Lymphoid cell analyses in REAR and FVB/N mice expressed as percentages of cells.

	Bone Marrow		Spleen		Lymph Node		Thymus	
	REAR	FVB/N	REAR	FVB/N	REAR	FVB/N	REAR	FVB/N
**CD4**	1.9 ± 0.3	1.9 ± 0.2	36.0 ± 0.2	43.7 ± 1.3	64.5 ± 2.4	45.5 ± 24.6	14.5 ± 1.3	11.4 ± 0.4
**CD8**	1.6 ± 0.5	2.5 ± 0.6	11.3 ± 0.6	11.9 ± 0.4	18.9 ± 1.3	19.1 ± 2.1	6.2 ± 0.6	6.0 ± 1.0
**CD4/CD8**	0 ± 0	0.1 ± 0	0.4 ± 0.1	0.3 ± 0.1	0.8 ± 0.2	7.3 ± 9.2	70 ± 4.5	80 ± 0.4
**CD44**	92 ± 0.7	91.7 ± 0.9	57.0 ± 0.7	48.1 ± 0.9	21.6 ± 0.7	35.4 ± 22	2.3 ± 0.6	1.4 ± 0.4
**CD4/CD44**	1.8 ± 0.3	1.8 ± 0.2	30.3 ± 0.3	32.7 ± 2.2	53.5 ± 0.7	43.0 ± 1.8	2.4 ± 0.6	2.2 ± 0.8
**CD8/CD44**	1.0 ± 0.3	1.5 ± 0.3	4.7 ± 0.2	3.9 ± 0.2	7.1 ± 0.7	17.1 ± 16.8	4.3 ± 1.0	3.9 ± 0.5
**IGM/B220**	32.1 ± 2.6	30.3 ± 2.5	32.1 ± 2.6	30.3 ± 2.5	2.4 ± 0.5	3.2 ± 0.0	0.2 ± 0.1	0.3 ± 0.1
**CD3/NK1.1**	0.6 ± 0.1	0.4 ± 0.1	1.0 ± 0.3	0.6 ± 0.1	0.4 ±0	0.4 ± 0	0.3 ±0.1	0.3 ± 0.1
**CD94/NK1.1**	0.1 ±0.1	0.1 ± 0.1	1.3 ± 0.3	1.8 ±0.6	0.9±0.0	0.6 ± 0.6	0 ±0	0 ± 0
**TCR αβ**	3.7 ± 1.5	5.8 ± 0.8	45.8 ± 6.9	49.6 ± 3.9	69.7 ± 14.7	73 ± 19.1	12.2 ± 10.6	33.8 ± 26.5
**TCR γδ**	0.7 ± 0.4	0.8 ± 0.1	1.3 ± 0.1	0.7 ± 0.5	1.3 ± 0.2	0.8 ± 0.4	2.1 ± 0.7	0.9 ± 0.6

### Tumor growth in REAR mice is inhibited by tumor cell vaccination

In order to assess whether REAR mice could mount an immunologic anti-tumor response to M6 cell tumors, a cell-based vaccination experiment was performed. Three cohorts of 6 eight-week old REAR mice were utilized. The first group of REAR mice was vaccinated with irradiated cells, injected intraperitoneally twice a week for two weeks with 3 x 10^6^ M6 cells exposed to 70 Gy of radiation (Mark-1 gamma-irradiator, JL Shepherd & Associates, San Fernando, CA). The following week 1 x 10^6^ M6 cells were implanted into the #4 mammary fat pad and tumor growth was monitored. The second group of mice was injected twice a week for two weeks with 3 x 10^6^ irradiated M6 cells expressing GM-CSF. The following week 1 x 10^6^ M6 cells were implanted into the #4 mammary fat pad. The third control group received no irradiated cells but was implanted with 1 x 10^6^ M6 at the same time as the other two groups. Tumor growth was monitored weekly.

**Fig 6:**
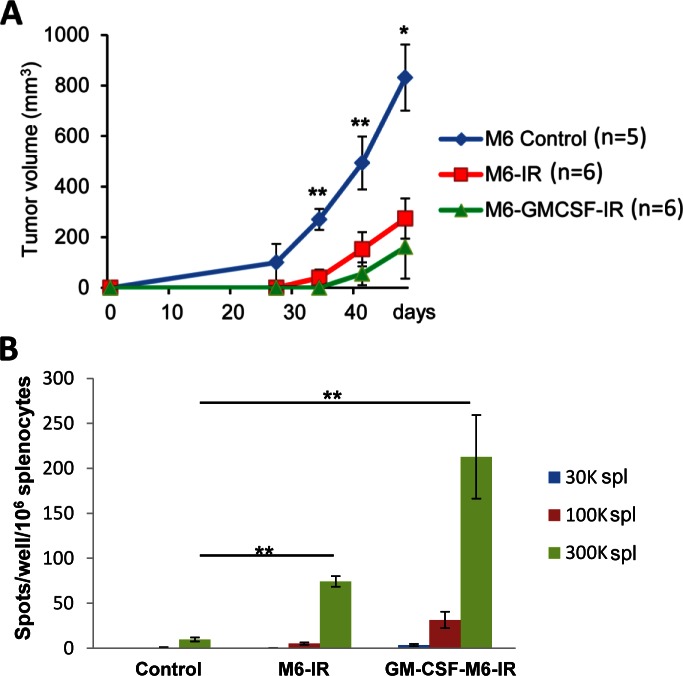
Vaccination of mice with irradiated M6 cells or irradiated M6 cells expressing GM-SCF results in reduced tumor volume and increased immunotoxicity of splenocytes. **(**A) Mice were given four injections of irradiated cells prior to mammary gland implantation of 10^6^ live M6 cells. Tumor volume was monitored over a 50 day period. (B) At the end point of experiment spleens of 2 mice from each group were used for splenocyte cytotoxicity measured by ELISPOT assay for different ratio of effector:target cells.

The vaccination of irradiated M6 cells prior to the implantation of live M6 tumor cells reduced tumor growth by almost 70% compared to the non-vaccinated control group (Fig 6A). Although tumor growth tended to be further inhibited in the mice pre-vaccinated with M6 cells expressing GM-CSF, this was not statistically significant compared the M6 irradiated cells.

In order to further confirm that the cell-based vaccinations induced an IFNγ response from lymphoid cells derived from the REAR mice in response to M6 tumor cells, an ELISPOT assay was performed on splenocytes derived from two mice from each of the experimental cohorts. As seen in Fig 6B, a minimal IFNγ response was observed from splenocytes obtained from the control mice, whereas splenocytes from mice treated with irradiated M6 or M6-GM-CSF cells had much stronger responses. There was a good negative correlation between tumor volume in the mice and the cytotoxic lymphocyte activity as demonstrated by a high R^2^ value of 0.7915 (S2 Fig). These results demonstrate that the cell vaccinations elicited a clear inhibitory immunologic response against tumor growth in the REAR mice.

## Discussion

Genetically engineered mouse models provide potent tools to address many critical issues in cancer biology. The ideal mouse cancer model for pre-clinical applications should recapitulate important genetic and molecular characteristics of the human disease, have a relatively short tumor latency, and a high tumor penetrance with the tumor evolving in the context of an intact immune system. Based upon the results of this study, the REAR mouse transplant system, therefore, is a novel model for metastatic basal-like triple-negative breast cancer.

This line of mice arose spontaneously within a C3(1)/Tag mouse colony [16]. Genetic analysis revealed that this subline of mice harbor a rearrangement of the original multiple tandem copies of the C3(1)/Tag transgene, leaving only one copy in the original chromosomal locus. Importantly, REAR mice do not develop the phenotypic abnormalities typical of the original C3(1)/Tag mice and are immune tolerant of SV40 Tag. When incidental lesions do develop, they tend to occur in older (>12mos) mice and are related to abnormalities commonly seen in aged FVB/N mice [31].

Of the lesions that we observed in REAR mice, only one leiomyosarcoma in a 24-month-old male animal stained positively for T-antigen by immunohistochemistry. Of interest, only one older mouse (out of 34 examined) developed a mammary tumor after 18 months of age. This is in contrast to the C3(1)/Tag mice, where 100% of female mice develop mammary tumors by 6 months of age [16]. Neither the primary tumor, nor the metastases stained positive for T-antigen by IHC. It is possible that the expression of T-antigen may be below the level of detection in this particular tumor or, more likely, tumor development is not due to the expression of T-antigen, as this tumor does not resemble a typical T-antigen driven malignancy but may have developed coincidentally or in response to a pituitary adenoma that commonly occur in aged FVB/N mice [33].

Since REAR mice have a fully functioning, intact immune system, they reject non-T-antigen driven tumors arising from non-syngeneic mice and human cell line xenografts, similar to wild-type FVB/N mice. A normal immune system is also evident in the comparative lymphoid profiles, which show no significant differences between REAR mice and wild-type FVB/N mice.

Importantly, C3(1)/Tag-REAR mice are tolerant to the implantation of SV40-Tag mammary and prostate tumors. When implanted with C3(1)/Tag mammary tumor derived M6 cells, female mice developed palpable tumors within 28 days (Fig 4A), approaching the maximum allowable tumor burden (2 cm in diameter) by 48 days post-implantation. In all cases, wild-type FVB/N mice failed to develop tumors. In addition, REAR mice are tolerant to another SV40-Tantigen derived tumor, as mice implanted with prostate tumor fragments from the TRAMP model develop palpable tumors within 7 weeks post transplantation.

Both fat pad and tail vein injection of M6 cells resulted in robust development of metastases. Lung and liver metastases were observed in 88% and 27% of mice implanted with M6 cells into the mammary fat pads. Tail vein injection of M6 cells into REAR mice resulted in 100% mortality by 12 weeks post-injection, with the mice developing distant metastases to multiple organs including the lungs (100%) and liver (67%). Importantly, 45% of the mice developed brain metastases, which is highly unusual for mouse models of breast cancer. Brain metastases often occur in patients with TNBC but few animal models exist to study breast cancer brain metastases [34, 35]. Most mouse models of brain metastasis for breast cancer involve intracardiac injection of brain-tropic cancer cells [36, 37]. Although such models are efficient at causing brain metastases, these models do not recapitulate the natural formation of brain metastases from primary tumors, potentially missing key requirements for the dissemination of cancer cells to distant sites.

The REAR mouse model will allow for the testing of therapies in the prevention and treatment of metastases, including CNS-metastases, as host immune functions are important for the elimination of potentially metastatic cells. Recapitulating the process of metastasis in an immune competent mouse model provides an opportunity to identify molecular mechanisms of tumor dissemination and new targets for therapies. For example, treatments with cell based vaccines, transfer of effector T cells incorporating chimeric receptors or manipulations of immune checkpoint regulators including CTLA-4 and PD1 could be explored to induce tumor rejection using this model. As a proof-of-principle, we demonstrated using a cell-based vaccination strategy that the REAR mice can illicit an immune response to the M6 tumors that correlates to the activation of IFNγ producing lymphoid cells in response to M6 cells. Therefore, the REAR model system should be very useful in preclinical studies combining standard therapies with novel immune therapy approaches.

## Supporting Information

S1 Fig Quantitative PCR analysis of Tag copy number in C3(1)/Tag mice compared to REAR mice. Tag copy number was normalized to albumin as described in the Methods section. Standard curve of PCR quantitation is seen in the left panel. C3(1)/Tag mice displayed about an 11:1 Tag/albumin ratio compared to REAR mice which displayed a ratio of about 2:1. Wild type FVB/N mice had not measurable levels of Tag as a control.
(TIF)

S2 Fig Mammary gland tumor volume inversely correlates with immunotoxicity of splenocytes in mice. Number of spots obtained by ELISPOT analysis for 300K effector cells per 50K target cells are plotted against tumor volume. A strong negative statistical correlation was observed between the tumor volume and lymphocyte cytotoxicity (R^2^ = 0.7915).
(TIF)

S1 Table Incidence of metastatic lesions in mammary fat pad xenografted Tag-REAR mice. (DOCX)

S2 Table Incidence of metastatic lesions in tail vein xenografted Tag-REAR mice. (DOCX)
