# Supplementary figures and images for: Development and Preclinical Application of an Immunocompetent Transplant Model of Basal Breast Cancer with Lung, Liver and Brain Metastases
Source: PLoS One. 2016 May 12;11(5):e0155262. doi: 10.1371/journal.pone.0155262 (PMC4865188; doi:10.1371/journal.pone.0155262)

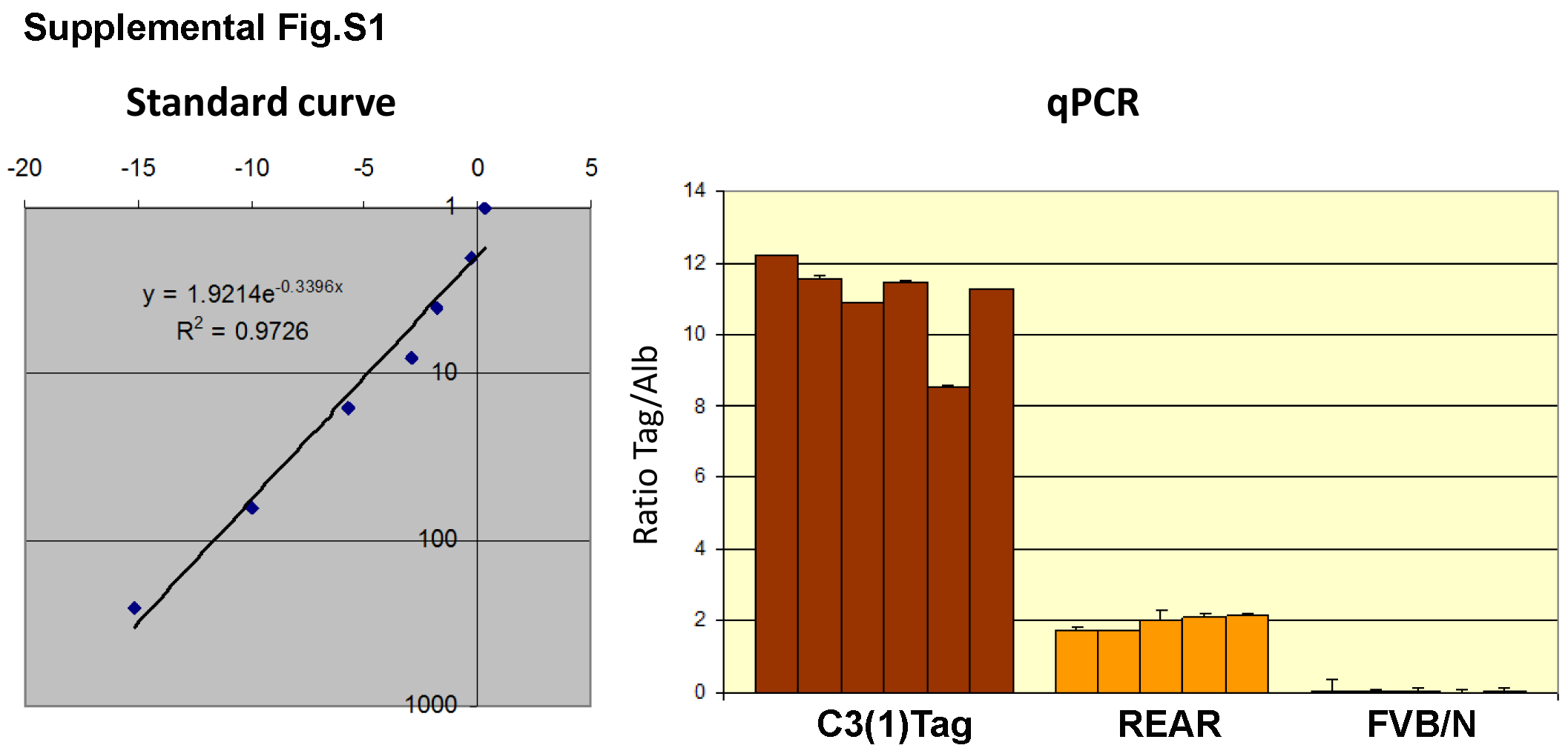

Supplement: S1 Fig — Tag copy number was normalized to albumin as described in the Methods section. Standard curve of PCR quantitation is seen in the left panel. C3(1)/Tag mice displayed about an 11:1 Tag/albumin ratio compared to REAR mice which displayed a ratio of about 2:1. Wild type FVB/N mice had not measurable levels of Tag as a control. (TIF) [file pone.0155262.s001.tif]

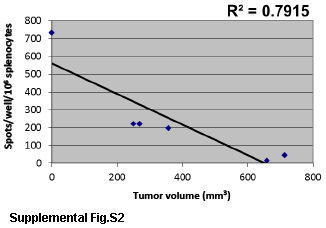

Supplement: S2 Fig — Number of spots obtained by ELISPOT analysis for 300K effector cells per 50K target cells are plotted against tumor volume. A strong negative statistical correlation was observed between the tumor volume and lymphocyte cytotoxicity (R2 = 0.7915). (TIF) [file pone.0155262.s002.tif]
