# Supplementary material for: Development and Preclinical Application of an Immunocompetent Transplant Model of Basal Breast Cancer with Lung, Liver and Brain Metastases
Source: PLoS One. 2016 May 12;11(5):e0155262. doi: 10.1371/journal.pone.0155262 (PMC4865188; doi:10.1371/journal.pone.0155262)
Supplement: S1 Table — (DOCX) [file pone.0155262.s003.docx]

| **Supplemental Table 1. - Incidence of metastatic lesions in mammary fat pad xenografted Tag-REAR mice.** | | | | | |
| --- | --- | --- | --- | --- | --- |
| Animal No | Lung | Liver | Spleen | Kidney | Brain |
| 705 | X | X |  | NC |  |
| 655 | X |  |  | NC |  |
| 653 | X |  |  | NC |  |
| 652 | X |  |  | NC |  |
| 649 | X |  |  | NC |  |
| 752 | X | X |  |  |  |
| 751 |  |  |  |  |  |
| 744 | X |  |  |  |  |
| 700 | X |  |  |  |  |
| 746 | X |  |  |  |  |
| 699 | X | X |  |  |  |
| 178 | X | NC | NC | NC | NC |
| 276 | X | X |  |  |  |
| 277 | X | X |  |  |  |
| 786 | X | X |  |  |  |
| 787 |  |  |  |  |  |
| Incidence | 14/16 (88%) | 4/15 (27%) | 0/15 | 0/10 | 0/15 |

LN = lymph node.

NC = not collected/not present in section.

Spleen data not shown (no metastases noted).
