# Supplementary material for: Development and Preclinical Application of an Immunocompetent Transplant Model of Basal Breast Cancer with Lung, Liver and Brain Metastases
Source: PLoS One. 2016 May 12;11(5):e0155262. doi: 10.1371/journal.pone.0155262 (PMC4865188; doi:10.1371/journal.pone.0155262)
Supplement: S2 Table — (DOCX) [file pone.0155262.s004.docx]

| **Supplemental Table 1. - Incidence of metastatic lesions in tail vein xenografted Tag-REAR mice.** | | | | | |
| --- | --- | --- | --- | --- | --- |
| Animal No. | Mammary | Lung | Liver | Kidney | Brain |
| 1574 | X (LN) | X | X |  |  |
| 1576 | X (LN) | X | X | X (LN) |  |
| 1579 | NC | X | X |  | NC |
| 1557 | NC | X |  | X |  |
| 1008 | NC | X |  |  | X |
| 1559 |  | X |  |  | X |
| 2541 |  | X | X |  |  |
| 2543 |  | X |  |  |  |
| 668 | NC | X | X | NC | X |
| 665 | NC | X | X | NC | X |
| 664 | NC | X | X | NC | X |
| 663 | NC | X | X | NC |  |
| Incidence | 2/5 (40%) | 12/12 (100%) | 8/12 (67%) | 2/8 (25%) | 5/11 (45%) |
| LN = lymph node.  NC = not collected/not present in section.  Spleen data not shown (no metastases noted). | | | | | |
